# Molecular Cloning and Characterization of Small Heat Shock Protein Genes in the Invasive Leaf Miner Fly, *Liriomyza trifolii*

**DOI:** 10.3390/genes10100775

**Published:** 2019-10-03

**Authors:** Ya-Wen Chang, Xiao-Xiang Zhang, Ming-Xing Lu, Yu-Zhou Du, Keyan Zhu-Salzman

**Affiliations:** 1 College of Horticulture and Plant Protection, Institute of Applied Entomology, Yangzhou University, Yangzhou 225009, China; changyawen@hotmail.com (Y.-W.C.); zxxyzu@yahoo.com (X.-X.Z.); lumx@yzu.edu.cn (M.-X.L.); 2 Joint International Research Laboratory of Agriculture and Agri-Product Safety, The Ministry of Education, Yangzhou University, Yangzhou 225009, China; 3 Department of Entomology, Texas A&M University, College Station, TX 77843, USA

**Keywords:** *Liriomyza trifolii*, small heat shock protein, development stage, thermal stress, expression pattern

## Abstract

Small heat shock proteins (sHSPs) comprise numerous proteins with diverse structure and function. As molecular chaperones, they play essential roles in various biological processes, especially under thermal stresses. In this study, we identified three sHSP-encoding genes, *LtHSP19.5*, *LtHSP20.8* and *LtHSP21.7b* from *Liriomyza trifolii*, an important insect pest of ornamental and vegetable crops worldwide. Putative proteins encoded by these genes all contain a conserved α-crystallin domain that is typical of the sHSP family. Their expression patterns during temperature stresses and at different insect development stages were studied by reverse-transcription quantitative PCR (RT-qPCR). In addition, the expression patterns were compared with those of *LtHSP21.3* and *LtHSP21.7*, two previously published sHSPs. When pupae were exposed to temperatures ranging from −20 to 45 °C for 1 h, all *LtsHSP*s were strongly induced by either heat or cold stresses, but the magnitude was lower under the low temperature range than high temperatures. Developmentally regulated differential expression was also detected, with pupae and prepupae featuring the highest expression of sHSPs. Results suggest that *LtsHSP*s play a role in the development of the invasive leaf miner fly and may facilitate insect adaptation to climate change.

## 1. Introduction

*Liriomyza trifolii* (Burgess) (Diptera: Agromyzidae) is an economically important and highly polyphagous pest in fields and greenhouses [1]. Both larvae and adults damage crop plants. The larvae tunnels in the leaves, whereas female adults puncture the leaf tissues for oviposition [2,3,4]. Originating from America, it has rapidly spread throughout the world [5]. In mainland China, *L. trifolii* was first recorded in Guangdong in 2005 [6], and now it has distributed to more than ten provinces [7]. *L. trifolii*, *L. sativae* and *L. huidobrensis* are the most important vegetable leaf mining pests in China [8,9,10]. *L. sativae* is the dominant species and has been detected throughout the country, whereas *L. huidobrensis* only occurs in regions of relatively high altitude [11,12]. *L. trifolii* mostly occurs in southeast coastal regions, but recently has been identified in certain northern provinces as well [7,13]. Temperature, particularly low temperature, appears to be the most important environmental factor that affects the distribution of *Liriomyza* species [8]. Some studies have been carried out to explore the response of *Liriomyza* species to temperature change and temperature-mediated interspecific competition [8,14,15,16]. 

Heat shock proteins (HSPs), including HSP90, HSP70, HSP60, HSP40 and small HSPs (sHSPs), are categorized based on molecular weight and sequence information [17,18,19,20]. In response to various stress factors, such as extreme temperatures, drought, osmotic stresses, heavy metals, oxidation, and UV radiation, organisms commonly synthesize HSPs [21,22,23,24]. Over-expression of HSPs has been commonly used as an indicator of insect tolerance to temperature stress [19,25]. Small HSPs have the greatest variation in structure and function among molecular chaperones, with molecular weight ranging from 12 to 43 kDa and a conserved α-crystallin domain [23]. They assist in the correct folding of nascent proteins, and prevent functional proteins from denaturation and aggregation induced by stresses [19,26,27,28]. sHSPs play an important role in increasing heat tolerance and protect organisms from thermal injury [29]. For instance, in the rice stem borer, *Chilo suppressalis*, *CsHSP19.8* and *CsHSP24.3* are both induced by high and low temperature extremes [30,31]. Similarly, expression of multiple sHSP genes in *Choristoneura fumiferana*, *Frankliniella occidentalis,* and *Bactrocera dorsalis* is significantly upregulated under thermal stresses [32,33,34]. sHSPs are also developmentally regulated in many insects [35]. sHSPs in *Drosophila melanogaster* are highly expressed during male gametogenesis [36] and embryonic development [37]. Some sHSPs in *Bombyx mori*, *B. dorsalis* and *Spodoptera litura* are induced by 20-hydroxyecdysone exposure, implying their involvement in metamorphosis [34,38,39,40]. 

sHSPs in closely related *L. sativae* and *L. huidobrensis* are induced by high and low temperature stresses [41]. In *L. sativae*, the expression of sHSPs during development has been investigated and the transcription of *LsHSP19.5*, *LsHSP20.8,* and *LsHSP21.7* peaks during the pupal stage [42]. In *L. trifolii,* the expression profile of *LtHSP21.3* has been shown to be similar to those of homologous genes in congeneric species under different temperature treatments [13]. When used to evaluate the stability of reference genes, expression of *LtHSP21.7* is shown to be induced by temperature stresses and is varied at different developmental stages [43].

In this study, to explore the diversity of structure and potential functions of sHSPs in *L. trifolii*, we identified three new sHSP cDNAs and analyzed their transcript profiles during temperature stresses and at different developmental stages. We compared their expression patterns with those of *LtHSP21.3* and *LtHSP21.7*, which have already been published. Our results provide new insight into physiological responses of *L. trifolii* under climate change that potentially influences the distribution of leaf miner flies.

## 2. Materials and Methods

### 2.1. Preparation of Insects of Different Developmental Stages

*L. trifolii*, originally collected from Yangzhou, China, was reared in the laboratory for more than 3 years at 26 ± 1 °C with a 16:8 h light:dark cycle [44]. Larvae and adults were reared on bean plants (*Vigna unguiculata*) and the leaves with tunnels were collected for pupation. The 3rd instar larvae, prepupae, 2-day- and 10-day-old pupae, and male and female adults were subjected to gene expression analysis. Three biological replicates were collected (n = 30). 

### 2.2. Temperature Treatments 

Two-day-old pupae (n = 30) were collected and placed into a water bath (DC-3010, Ningbo, China) and exposed for 1 h at low temperatures of −20, −17.5, –15, −12.5, −10, −7.5, −5, −2.5, 0, and 2.5 °C, and high temperatures of 27.5, 30, 32.5, 35, 37.5, 40, 42.5, and 45 °C. Pupae used for control were maintained at 25 °C. After exposure to various temperatures, pupae were incubated at 25 °C for 1 h, followed by instant freezing in liquid nitrogen, and storage at −80 °C. Each treatment was repeated four times.

### 2.3. RNA Isolation and Cloning Experiments 

Total RNA was extracted using the SV Total RNA Isolation System (Promega, Fitchburg, WI, USA) and treated with DNase I to eliminate DNA contamination, following the manufacturer’s protocol. Integrity and purity of RNA was determined by agarose gel electrophoresis and spectrophotometry (Eppendorf Bio Photometer plus, Hamburg, Germany). First-strand cDNA was synthesized from 1 μg of total RNA using the First Strand cDNA Synthesis Kit (Fermentas, Ontario, Canada). Three putative sHSP genes, selected based on the analysis of our unpublished transcriptome data, were PCR amplified with gene-specific primers (Table S1). Touchdown PCR conditions were as follows: 94 °C for 3 min, 19 cycles of 94 °C for 30 s, 65–45 °C (annealing temperature decreased by 1 °C /cycle, from 65 °C to a “touchdown” 45 °C) for 30 s, 72 °C for 1 min, and then 25 cycles of 94 °C for 30 s, 45 °C (annealing temperature) for 30 s, and 72 °C for 1 min, followed by extension at 72 °C for 10 min. 5’ and 3’ rapid amplification of cDNA ends (5’ and 3’ RACE) were performed to obtain full-length cDNAs using a SMART RACE cDNA Amplification Kit (Clontech, CA, USA) according to the manufacturer’s instructions. LA Taq DNA Polymerase (Takara, Japan) was used for the PCR amplification and PCR parameters were as follows: 94 °C for 3 min, 35 cycles of 94 °C for 30 s, 68 °C for 30 s, and 72 °C for 3 min, followed by extension at 72 °C for 10 min. After obtaining the sequence information of the three sHSP genes, specific primers were designed to amplify the full-length of cDNAs. Gene-specific primers are shown in Table S1. The full-length cDNAs were purified using a gel extraction kit (Axygen, New York, NY, USA), cloned into gam-T Easy Vector (Promega, Fitchburg, WI, USA) and subjected to sequencing.

### 2.4. Reverse Transcription Quantitative PCR

Total RNA (0.5 μg) was reverse-transcribed into first-strand cDNA using the Bio-Rad iScript™ cDNA Synthesis Kit (Bio-Rad, CA, USA). The RT-qPCR reactions were performed using a CFX96 Real-Time PCR System (Bio-Rad Laboratories, Berkeley, CA, USA) in 20 μL reaction volume, as previously described [13]. The relative quantifications of *LtsHSP*s were assessed using the 2^−^^ΔΔCt^ method [45] and *ACTIN* was used as a reference gene, because it is commonly used and the most optimal reference gene in *L. trifolii* under different experimental conditions [43]. Each sample was assessed in triplicate (technical replicates).

### 2.5. Sequence Alignment of sHSPs and Data Analysis

Full-length cDNA sequences of the three *LtsHSP*s were used as queries to search for other homologous sHSPs using the BLAST programs (http://www.ncbi.nlm.gov/BLAST/). Sequence alignments were conducted using Clustal X software [46], and the open reading frames (ORFs) were identified with ORF Finder (https://www.ncbi.nlm.nih.gov/orffinder/). The deduced amino acid sequences of sHSPs were analyzed by ExPASy (Swiss Institute of Bioinformatics, Switzerland). The phylogenetic relationship of sHSPs was generated by MEGA 6.0 [47], using a neighbor joining (NJ) method based on the Poisson correction model with a bootstrap value of 1000. The protein structure of sHSP genes was predicted by the SWISS-MODEL (https://www.swissmodel.expasy.org/).

Data were analyzed with one-way ANOVA, followed by Tukey’s multiple comparison and analysis with SPSS v. 16.0 (SPSS, Chicago, IL, USA). For ANOVA, data were transformed for homogeneity of variance tests. Differences were considered statistically significant when *P <* 0.05.

## 3. Results

### 3.1. Cloning, Characterization and Phylogenetic Analysis of Three sHSP Genes from Liriomyza trifolii

**Figure 1:**
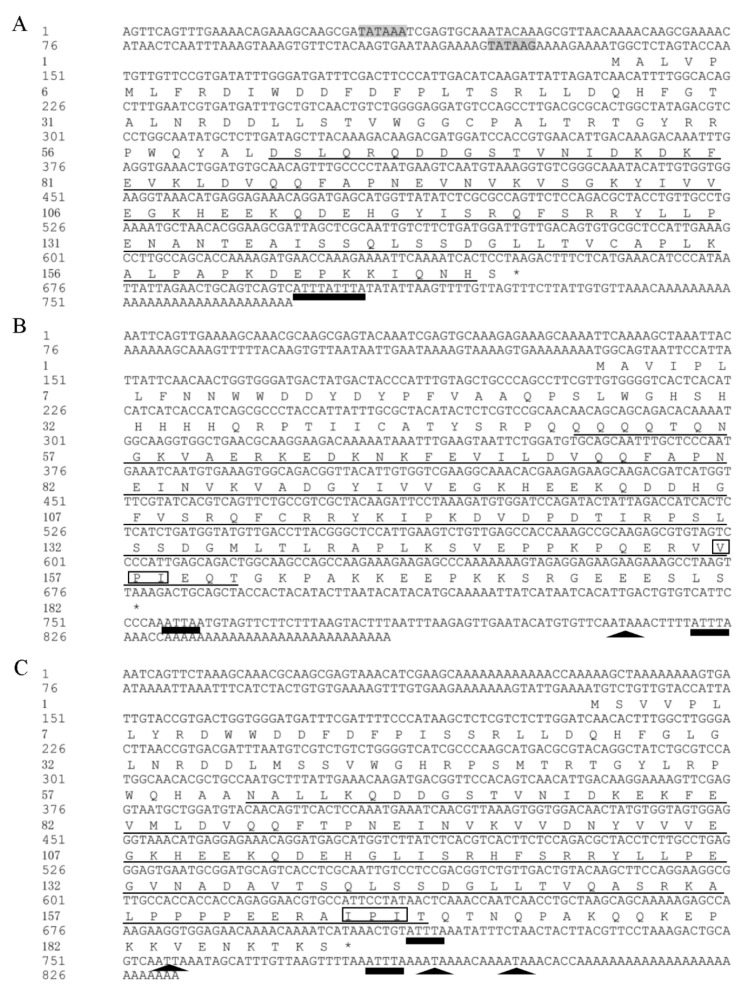
Nucleotide sequences for *L. trifolii* sHSP cDNAs and their predicted amino acid sequences. Nucleotide numbering starts with the adenine in the first methionine codon of the putative open reading frame. The highly conserved region, α-crystallin domain, is underlined. The asterisk indicates the translational termination codon. The putative polyadenylation signal is black triangles, the AT-rich element is black rectangles, the V/P/I motif is boxed and the TA-rich regions are indicated by shading. sHSP = small heat shock proteins.

**Figure 2:**
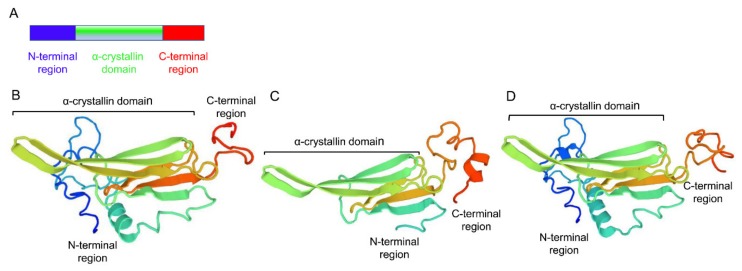
Structure of sHSPs. (**A**) The domain organization of sHSPs: N-terminal region (blue), α-crystallin domain (turquoise), and the C-terminal region (red); (**B**–**D**) protein tertiary structure of *LtHSP19.5* (**B**); *LtHSP20.8* (**C**); and *LtHSP21.7b* (**D**).

Three sHSPs, namely *LtHSP19.5*, *LtHSP20.8*, and *LtHSP21.7b* (GenBank accession nos. MG195951, MG195952, and MG195953, respectively), were cloned from *L. trifolii*. The ORFs were 516, 546, and 573 bp long, encoding proteins with 171, 181, and 190 amino acids, molecular weights of 19.46, 20.89, and 21.72 kDa, and isoelectric points of 5.56, 6.71, and 6.07, respectively, as seen in Table 1. Their deduced amino acid sequences contained a typical α-crystalline domain. *LtHSP20.8* and *LtHSP21.7b* contained V/P/I and I/P/I motifs near the C-terminus, respectively, as seen in Figure 1. The three-dimensional (3D) structures of sHSP genes were predicted by the SWISS-MODEL website using the human αB-crystallin (PDB 2yjdA) as the best template, and it shares 43.23, 54.05 and 44.17% sequence identity with *LtHSP19.5, LtHSP20.8, LtHSP21.7b,* respectively, as seen in Figure 2. The deduced 3D structures of sHSPs share typical features of the sHSP family.

Two TA-rich regions (TATA) were identified in the 5’UTRs of *LtHSP19.5*, but not in *LtHSP20.8*, and *LtHSP21.7b*. The 3’UTRs of these sHSPs also contain two typical motifs. AT-rich elements (ATTTA) were found in 3’UTRs of all three sHSPs, whereas polyadenylation signal elements (AATAAA or ATTAAA) were only found in 3’UTRs of *LtHSP20.8* and *LtHSP21.7b,* as seen in Figure 1. 

**Figure 3:**
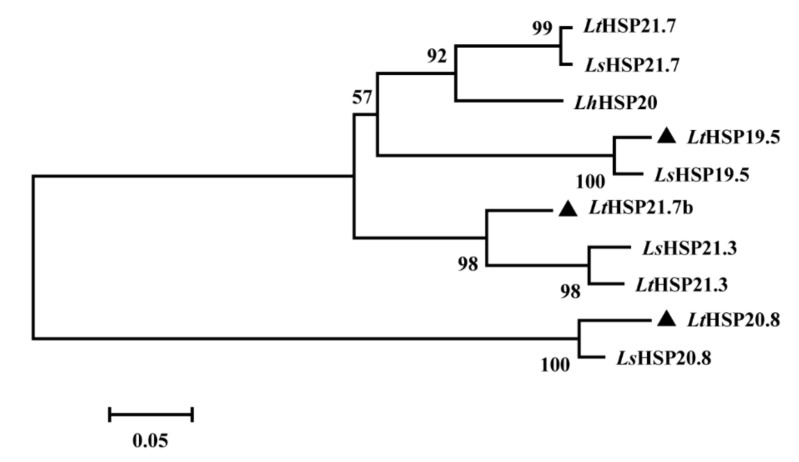
Neighbor-joining phylogenetic tree of *Liriomyza* sHSPs. Those three new *L. trifolii* sHSPs of this study are labeled with triangles. Numbers on the branches are the bootstrap values obtained from 1000 replicates (only bootstrap values >50 are shown). Abbreviations, species, and accession numbers include *LtHSP21.7* (*L. trifolii*, KY558641); *LsHSP21.7* (*L. sativae*, DQ452372); *LhHSP20* (*L. huidobrensis*, DQ452370); *LtHSP19.5* (*L. trifolii*, MG195951); *LsHSP19.5* (*L. sativae*, DQ452373); *LtHSP21.7b* (*L. trifolii*, MG195953); *LsHSP21.3* (*L. sativae*, DQ452371); *LtHSP21.3* (*L. trifolii*, KY231145); *LtHSP20.8* (*L. trifolii*, MG195952); and *LsHSP20.8* (*L. sativae*, DQ452374).

To examine the phylogenetic relationships between various sHSPs, the phylogenetic tree was generated using ten full-length sHSP family members, selected from three congener *Liriomyza* species using the neighbor-joining method. *LtHSP20.8* was grouped with *LsHSP20.8* in a separate branch*,* and *LtHSP19.5* and *LtHSP21.7b* were clustered into a larger group that contained several other orthologs genes, as seen in Figure 3.

### 3.2. Expression of Three LtsHSPs in Response to Temperature Treatments

**Figure 4:**
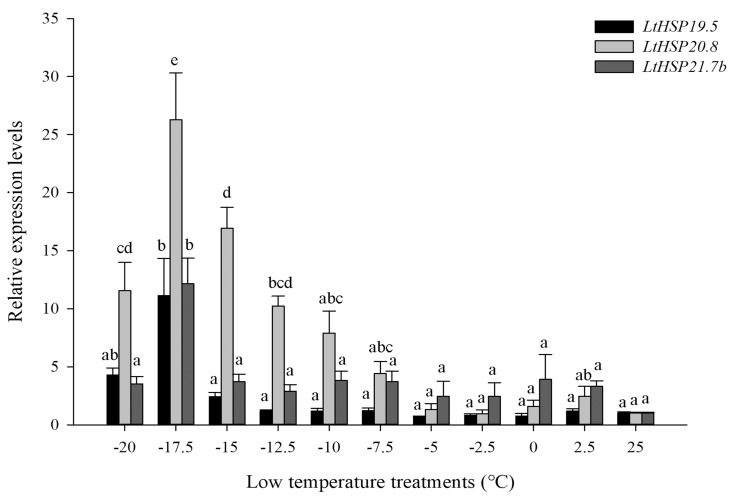
Relative expression levels of *LtHSP19.5, LtHSP20.8,* and *LtHSP21.7b* under low temperature treatments. The relative level of HSP expression represented the fold increase as compared with the expression in controls. The data were denoted as mean ± SE. One-way analysis of variance (ANOVA) was used to analyze the relative expression levels of three sHSPs under low temperature treatments. For the ANOVA, data were tested for homogeneity of variances and normality. Different lowercase letters indicate significant differences among different temperature treatments. Tukey’s multiple range test was used for pairwise comparison for mean separation (*P* < 0.05).

The relative mRNA levels of the three sHSPs were observed at different temperature stresses. In cold stress, compared with the control group at 25 °C, the expression level of three *LtsHSP*s were significantly increased after low temperature treatment (*LtHSP19.5*: *F*_10, 33_ = 23.514, *P <* 0.001; *LtHSP20.8*: *F*_10, 33_ = 29.116, *P* < 0.001; and *LtHSP21.7b*: *F*_10, 33_ = 4.245, *P* < 0.05). Gene expression peaked at –17.5 °C, which were 11.12-, 26.27- and 12.16-fold increase relative to the control, as seen in Figure 4.

**Figure 5:**
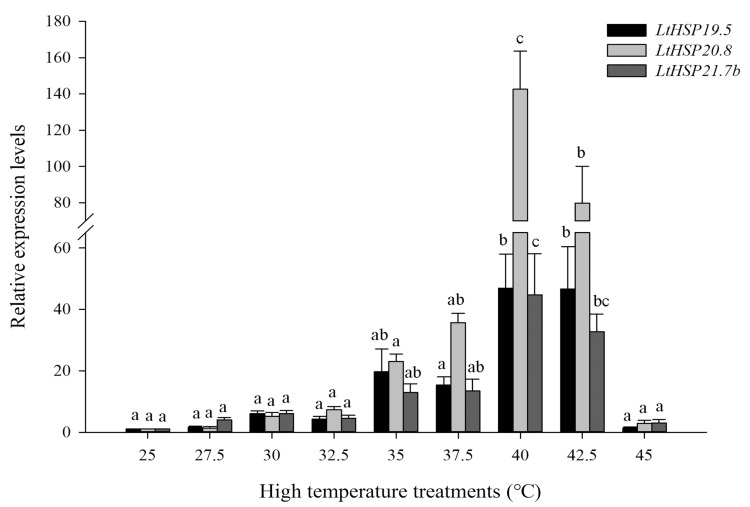
Relative expression levels of *LtHSP19.5, LtHSP20.8, and LtHSP21.7b* under high temperature treatments. The relative level of HSP expression represented the fold increase as compared with the expression in controls. The data were denoted as mean ± SE. One-way analysis of variance (ANOVA) was used to analyze the relative expression levels of three sHSPs under high temperature treatments. For the ANOVA, data were tested for homogeneity of variances and normality. Different lowercase letters indicate significant differences among different temperature treatments. Tukey’s multiple range test was used for pairwise comparison for mean separation (*P* < 0.05).

In heat stress, compared with the control group at 25 °C, expression of *LtsHSP*s were also significantly increased (*LtHSP19.5*: *F*_8, 27_ = 31.087, *P* < 0.001; *LtHSP20.8*: *F*_8, 27_ = 51.124, *P* < 0.001; and *LtHSP21.7b*: *F*_8, 27_ = 26.267, *P* < 0.001). The highest expression of *LtsHSP*s occurred at 40 °C, which were 46.93-, 142.56-, and 44.71-fold higher than that of control, respectively, as seen in Figure 5. 

### 3.3. Expression of Three LtsHSPs at the Developmental Stages

**Figure 6:**
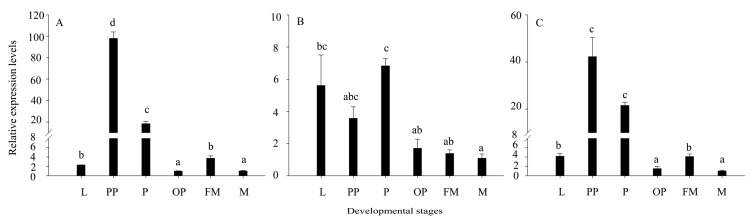
Relative expression levels of *LtHSP19.5*, *LtHSP20.8*, and *LtHSP21.7b* in different developmental stages of *L. trifolii*. The relative level of HSP expression represented the fold increase as compared with the expression in controls. (**A**) Relative expression levels of LtHSP19.5; (**B**) relative expression levels of *LtHSP20.8;* and (**C**) relative expression levels of *LtHSP21.7b*. The data were denoted as mean ± SE. One-way analysis of variance (ANOVA) was used to analyze the relative expression levels of three sHSPs in different developmental stages. For the ANOVA, data were tested for homogeneity of variances and normality. Different lowercase letters indicate significant differences among different developmental stages. Tukey’s multiple range test was used for pairwise comparison for mean separation (*P* < 0.05). Abbreviations: FM = females adult; M = males adult; L = third instar larvae; PP = prepupae; P = two-day-old pupae; and OP = ten-day-old pupae.

We investigated the mRNA level of three s*HSP*s through the developmental stage of *L. trifolii*, including 3rd instar larvae, prepupae, 2-day- and 10-day-old pupae, and male and female adults. All three *LtsHSP*s showed expression variations throughout the developmental stages. The expression of *LtHSP19.5* and *LtHSP21.7b* peaked at prepupae, which were significantly up-regulated by 98.10- and 42.27-fold relative to the control-male adults (*LtHSP19.5*: *F*_5, 12_ = 338.747, *P* < 0.001; *LtHSP21.7b*: *F*_5,12_ = 54.757, *P* < 0.001). Expression of *LtHSP20.8* peaked at 2-day-old pupae (up-regulated by 6.83-fold; *F*_5, 12_ = 6.640, *P* < 0.05) as seen in Figure 6.

### 3.4. Comparative Characteristics and Expression Pattern of Five LtsHSPs

We have previously characterized the expression pattern of *LtHSP21.3* under temperature stresses [13]. In this study, for comparison, the expression pattern of *LtHSP21.3* was also investigated at different developmental stages. As seen in Figure S1, the highest expression stage of *LtHSP21.3* occurred at 2-day-old pupal stages (*F*_5, 12_ = 13.935, *P* < 0.001), similar to that of *LtHSP20.8,* but different from other sHSPs in *L. trifolii*. 

Previously, the expression pattern of *LtHSP21.7* was studied under different experimental conditions for reference gene selection [43]. In this study, for comparison, the expression pattern of *LtHSP21.7* was also investigated at different temperature stresses (low temperatures: –20, −17.5, −15, −12.5, −10, −7.5, −5, −2.5, 0 and 2.5 °C; high temperature: 27.5, 30, 32.5, 35, 37.5, 40, 42.5 and 45 °C). The highest expression temperatures of *LtHSP21.7* was at −17.5 °C for low temperature stress (*F*_10, 33_ = 16.577, *P* < 0.001) as seen in Figure S1, and at 42.5 ℃ for high temperature stress (*F*_8, 27_ = 33.221, *P* < 0.001) also seen in Figure S1. Expression fold changes of *LtHSP21.7* under temperature stress were significantly lower than those of the other sHSPs in *L. trifolii*. The sequence characteristics of the five *LtsHSP*s were detailed in Table 1.

## 4. Discussion

In this study, three new sHSP-encoding genes were cloned from *L. trifolii*. Sequence analysis shows that all three predicted protein sequences contain the characteristic α-crystalline domain. In addition, *LtHSP20.8* and *LtHSP21.7b* also contained V/IXI/V motif. The propensity of the IPI/V motif to form multiple inter-subunit interactions may contribute to the diversity in structure and function seen in the α-crystallin [27]. Moreover, the 3’UTRs of *LtsHSP*s contain several other typical motifs, such as the poly adenylation signal (AATAAA or ATTAAA) [48] and the AT-rich element (ATTTA), which have been shown to afford greater mRNA stability and to contribute to the maintenance and re-establishment of basal levels of gene expression [42,49,50,51]. The number of TA-rich regions in 5’UTR of those three *LtsHSPs* were different; only two TA-rich regions were found in the 5’UTR of *LtHSP19.5,* but it was lacking in *LtHSP20.8* and *LtHSP21.7b.* The difference in the number of these elements have also been found in HSPs of other insect species [41], and the number of these elements may be related to the expression pattern of HSPs [52,53,54]. The phylogenetic analysis showed that *LtHSP19.5* and *LtHSP20.8* clustered with representatives of their orthologs, but *LtHSP21.7b* was clustered with *HSP21.3.* This clustering pattern was also recorded in previous studies [23,55], which suggested that the evolution of sHSP may be complex. However, the available sHSP data are limited, and the systematic illustration of the evolution of sHSPs is expected to be achieved through the genome-wide analysis in future.

In this study, three new *LtsHSPs* and two previously identified *Lt*s*HSPs* are all significantly up-regulated by low and high temperature treatments. The same expression patterns have also been observed in most previously recorded responses of sHSPs [30,56,57,58]. The sensitivity to temperature stresses of five *Lt*s*HSPs* was different but the temperatures of the maximal (*T*max) expression were comparable. The same highest expression temperature range suggests similar functionality under temperature stresses, except for *LtHSP21.7* at high temperatures. In this study, the response level of three sHSPs to different temperature stresses was higher than that of two published sHSPs (*LtHSP21.3* and *LtHSP21.7*), which was reflected by a higher gene expression fold. The five *LtsHSPs* varied in terms of temperature sensitivity and suggest a synergistic effect of different sHSP family members with respect to thermal tolerance, which is consistent with recent research of *LtHSP70s* [59]. Several sHSPs play a role in temperature stress together, and similar expression patterns were also found in *Chilo suppressalis* and *Bemisia tabaci* [31,60]. At the same time, it is worth noting that, like in cases of other HSPs, expression level of *LtHSPs* induced by high temperatures is higher than that induced by low temperatures. In addition, other mechanisms for confronting with the stress may play a role in resisting extreme low temperature stress, such as antioxidation and supercooling phenomenon.

Attention has been paid to the role of HSPs in the regulation of insect development [35]. It seemed that all three new *Lt*s*HSPs* and two previous *Lt*s*HSPs* could be expressed in all developmental stages in *L. trifolii.* However, all of the five sHSPs’ expression levels were significantly different in the developmental stages, and the expression fold of *LtHSP19.5, LtHSP21.3,* and *LtHSP21.7* [43] in prepupae was significantly different from that in control, while *LtHSP20.8* and *LtHSP21.7b* reached a peak in the pupal stage. For prepupae, the mature larvae just leave the leaves for pupation, so it may lead to more sensitivity to the environment temperature. Meanwhile, the expression level increased significantly during the transition from larvae to pupae, which is the same as the one observed in *LtHSP70s* [59]. This suggests that metamorphosis itself can serve as a factor to induce the expression of HSPs. The metamorphosis has large physical changes and HSPs, as important molecular chaperones, are involved in processes such as assembling, removing, folding, and refolding of different kinds of proteins [19]. Thus, the change of protein structure may lead to a relatively high expression level of HSPs [42]. 

## 5. Conclusions

In summary, genes encoding sHSPs of *L. trifolii* contained several typical conserved domain and motifs. Those *Lt*s*HSPs* could be significantly induced by temperature stresses and expressed during different stages of insect development. This study provides further insights into physiological responses of *L. trifolii* under climate change, and the mechanisms of distribution of leaf miner flies in response to temperature.

## Figures and Tables

**Table 1:** The Characteristics of Five sHSPs in *L. trifolii.*

Name of Genes	Molecular Weight	Isoelectric Point	Developmental Stages (Highest Expression)	High Temperature (*T*max)	Low Temperature (*T*max)
*Lt* *HSP19.5*	19.46 kDa	5.56	prepupae	40 °C	−17.5 °C
*Lt* *HSP20.8*	20.89 kDa	6.71	two-day-old pupae	40 °C	−17.5 °C
*Lt* *HSP21.7b*	21.72 kDa	6.07	two-day-old pupae	40 °C	−17.5 °C
*Lt* *HSP21.3*	21.23 kDa *	6.38 *	prepupae	40 °C *	−17.5 °C *
*Lt* *HSP21.7*	21.66 kDa *	7.03 *	prepupae *	42.5 °C	−17.5 °C

* Data of gene characteristics for *HSP21.3* and *HSP21.7* were obtained from Chang et al. (2017a, 2017b).

## Supplementary Materials

The following are available online at https://www.mdpi.com/2073-4425/10/10/775/s1, Figure S1: Supplementary relative expression levels of *LtHSP21.3* and *LtHSP21.7* at different developmental stages or under temperature treatments. The relative level of HSP expression represented the fold increase as compared with the expression in controls. (**A**) Relative expression levels of *LtHSP21.3* in different developmental stages; (**B**) Relative expression levels of *LtHSP21.7* under low temperatures; and (**C**) Relative expression levels of *LtHSP21.7* under high temperatures. The data were denoted as mean ± SE. One-way analysis of variance (ANOVA) was used to analyze the relative expression levels of three sHSPs in different developmental stages and under temperature treatments. For the ANOVA, data were tested for homogeneity of variances and normality. Different lowercase letters indicate significant differences among different temperature treatments. Tukey’s multiple range test was used for pairwise comparison for mean separation (*P* < 0.05). Abbreviations: FM = females adult; M: males adult; L: third instar larvae; PP: prepupae; P: two-day-old pupae; OP: ten-day-old pupae, Table S1: Primers used in the cDNA cloning and real-time quantitative PCR.
