# Supplementary material for: Molecular Cloning and Characterization of Small Heat Shock Protein Genes in the Invasive Leaf Miner Fly, Liriomyza trifolii
Source: Genes (Basel). 2019 Oct 3;10(10):775. doi: 10.3390/genes10100775 (PMC6826454; doi:10.3390/genes10100775)
Supplement: Supplementary file 1 [file genes-10-00775-s001.zip › Table S1.docx]

**Table S1.** Primers used in the cDNA cloning and real-time quantitative PCR

| **Gene** | | **Primer sequences(5’→3’)** | **Fragment length (bp)** | |
| --- | --- | --- | --- | --- |
| Primers for cDNA cloning and full-length cDNA amplification | | | | |
| *HSP19.5* | F | AATGTTGTTCCGTGATATTTGGG | | 483 |
|  | R | TTCTTTGGTTCATCTTTTGGTGC | |  |
|  | 5’ | TCACCAGCAGTGGACTTGACCTCG | | 280 |
|  | 3’ | AGTTCTCCAGACGCTACCTGTTGCC | | 273 |
|  | Full-length-F | ACAAAACAAGCGAAAACATAACTC | | 615 |
|  | Full-length-R | ATGGGATGTTTCATGAGAAAGTCT | |  |
| *HSP20.8* | F | ACAACAGCAGCAGACACAAAATG | | 215 |
|  | R | CTTTAGGAATCTTGTAGCGACGG | |  |
|  | 5’ | GCTGCTGTTGTTGCGGACGAGAGTA | | 297 |
|  | 3’ | AAAGCCGCAAGAGCGTGTAGTCCC | | 280 |
|  | Full-length-F | GTACAAATCGAGTGCAAAGAGAAA | | 743 |
|  | Full-length-R | GAAGAACTACATTAATTTGGGGAA | |  |
| *HSP21.7b* | F | GCTTTATTGAAACAAGATGACGG | | 341 |
|  | R | TTAGCAGGTTGATTGGTTTGAGT | |  |
|  | 5’ | GCAAGAGGTAGCGTCTGGAGAAGTGA | | 528 |
|  | 3’ | ACTTCTCCAGACGCTACCTCTTGCC | | 336 |
|  | Full-length-F | AATCAGTTCTAAAGCAAACGCAAG | | 767 |
|  | Full-length-R | AATGCTATTTAATTGACTGCAGTCTTT | |  |
|  | | | | |
| Primers for qRT-PCR | | | | |
| *HSP19.5* | F | GCTACCTGTTGCCTGAAAATGCT | | 118 |
|  | R | CTTTGGTTCATCTTTTGGTGCTG | |  |
|  |  |  | |  |
| *HSP20.8* | F | AACAACTGGTGGGATGACTATGAC | | 144 |
|  | R | CATTTTGTGTCTGCTGCTGTTGT | |  |
|  |  |  | |  |
| *HSP21.7b* | F | CTCCAGACGCTACCTCTTGCCT | | 159 |
|  | R | CTTAGCAGGTTGATTGGTTTGAGT | |  |
|  |  |  | |  |
| *HSP21.3* | F | GAAATCAATGTGAAAGTGGTGGA | | 175 |
|  | R | GAACCTTCAACAAGCCATCAGAT | |  |
|  |  |  | |  |
| *HSP21.7* | F | CAACAGTTTGCTCCCAATGAAG | | 125 |
|  | R | GAGGTAGCGTCTGGAGAAGTGA | |  |
|  |  |  | |  |
| *ACTIN* | F | TTGTATTGGACTCTGGTGACGG | | 73 |
|  | R | GATAGCGTGAGGCAAAGCATAA | |  |

Abbreviations: F, forward; R, reverse; 5’, 5’ RACE primer; 3’, 3’ RACE primer; ORF-F, genome amplification forward primer; ORF-R, genome amplification reverse primer.
