# Supplementary material for: Molecular Cloning and Characterization of Small Heat Shock Protein Genes in the Invasive Leaf Miner Fly, Liriomyza trifolii
Source: Genes (Basel). 2019 Oct 3;10(10):775. doi: 10.3390/genes10100775 (PMC6826454; doi:10.3390/genes10100775)
Supplement: Supplementary file 1 [file genes-10-00775-s001.zip › Figure S1.docx]

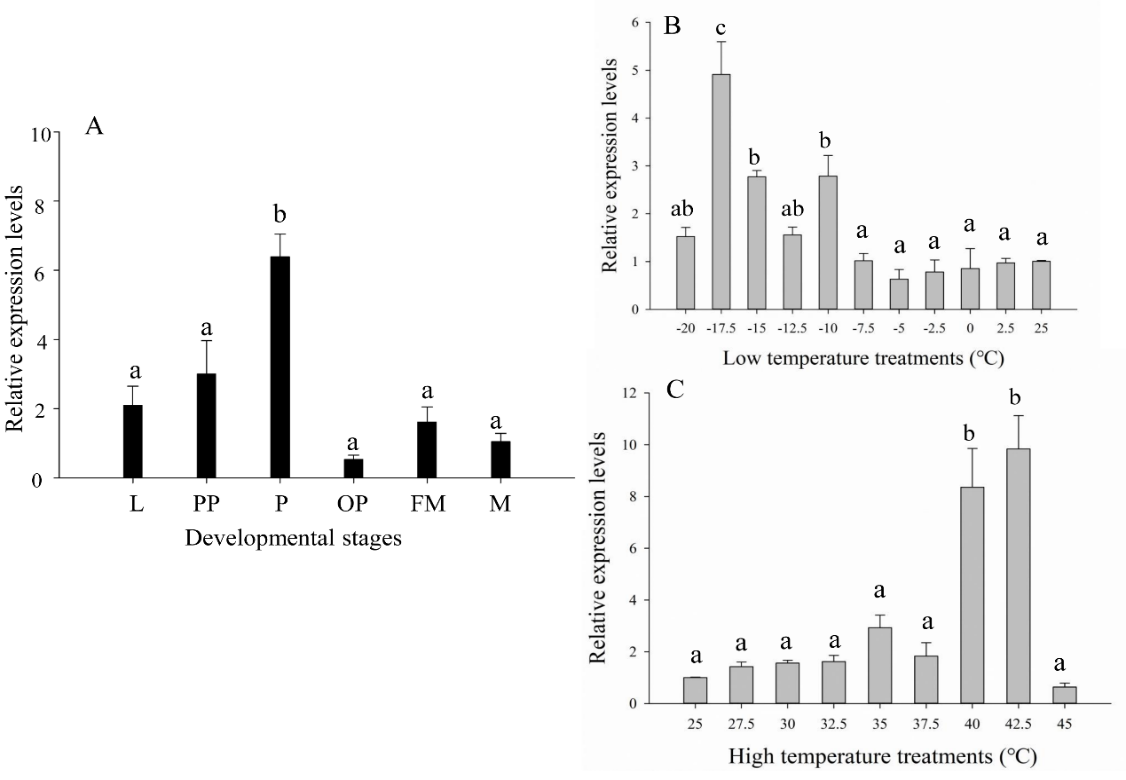


**Figure S1.** Supplementary relative expression levels of *LtHSP21.3* and *LtHSP21.7* at different developmental stages or under temperature treatments. The relative level of HSP expression represented the fold increase as compared with the expression in controls. (**A**) Relative expression levels of *LtHSP21.3* in different developmental stages; (**B**) Relative expression levels of *LtHSP21.7* under low temperatures; and (**C**) Relative expression levels of *LtHSP21.7* under high temperatures. The data were denoted as mean ± SE. One-way analysis of variance (ANOVA) was used to analyze the relative expression levels of three sHSPs in different developmental stages and under temperature treatments. For the ANOVA, data were tested for homogeneity of variances and normality. Different lowercase letters indicate significant differences among different temperature treatments. Tukey’s multiple range test was used for pairwise comparison for mean separation (*P* < 0.05). Abbreviations: FM= females adult; M: males adult; L: third instar larvae; PP: prepupae; P: two-day-old pupae; OP: ten-day-old pupae.
