# Supplementary figures and images for: Molecular Cloning and Characterization of Small Heat Shock Protein Genes in the Invasive Leaf Miner Fly, Liriomyza trifolii
Source: Genes (Basel). 2019 Oct 3;10(10):775. doi: 10.3390/genes10100775 (PMC6826454; doi:10.3390/genes10100775)

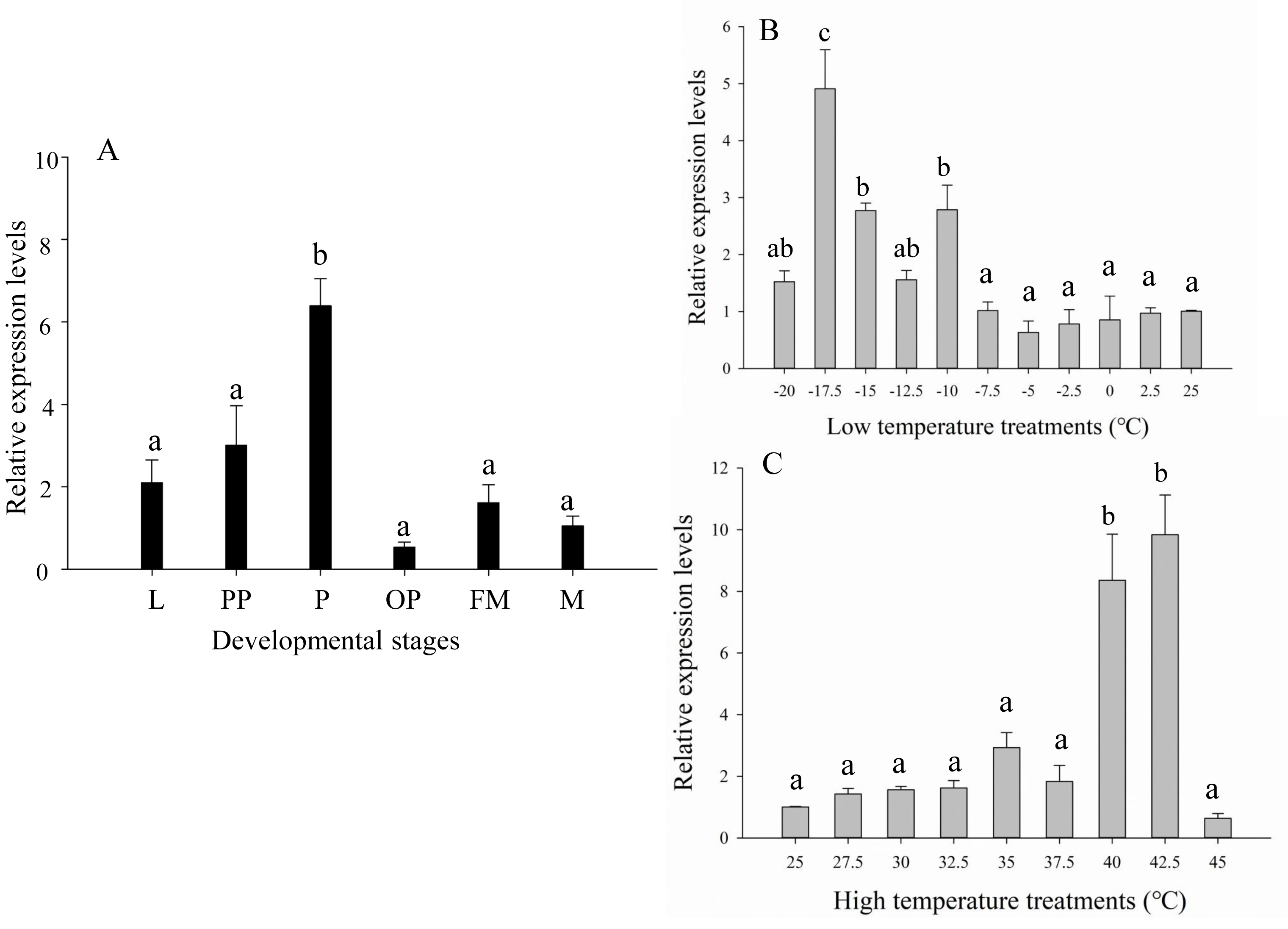

Supplement: Supplementary file 1 [file genes-10-00775-s001.zip › Figure S1.tif]
